# Antioxidant and anti-inflammatory activities of selected Chinese medicinal plants and their relation with antioxidant content

**DOI:** 10.1186/1472-6882-12-173

**Published:** 2012-10-06

**Authors:** Anjaneya S Ravipati, Lin Zhang, Sundar Rao Koyyalamudi, Sang Chul Jeong, Narsimha Reddy, John Bartlett, Paul T Smith, Kirubakaran Shanmugam, Gerald Münch, Ming Jie Wu, Manavalan Satyanarayanan, Balaram Vysetti

**Affiliations:** 1 School of Science and Health, Locked Bag 1797, Penrith South DC, NSW 1797, Australia; 2 Centre for Complementary Medicine Research, Locked Bag 1797, Penrith South DC, NSW 1797, Australia; 3 School of Medicine, University of Western Sydney, Locked Bag 1797, Penrith South DC, NSW 1797, Australia; 4 School of Pharmacy and Molecular Sciences, James Cook University, Townsville, Queensland, 4811, Australia; 5 CSIR-National Geophysical Research Institute, Uppal Road, Hyderabad, 500007, India

**Keywords:** Antioxidant activities, Anti-inflammatory properties, Phenolics, Flavonoids, Trace metals

## Abstract

**Background:**

The main aim of this study is to evaluate the antioxidant and anti-inflammatory properties of forty four traditional Chinese medicinal herbal extracts and to examine these activities in relation to their antioxidant content.

**Methods:**

The antioxidant activities were investigated using DPPH radical scavenging method and yeast model. The anti-inflammatory properties of the herbal extracts were evaluated by measuring their ability to inhibit the production of nitric oxide and TNF-α in RAW 264.7 macrophages activated by LPS and IFN- γ, respectively. The cytotoxic effects of the herbal extracts were determined by Alomar Blue assay by measuring cell viability. In order to understand the variation of antioxidant activities of herbal extracts with their antioxidant contents, the total phenolics, total flavonoids and trace metal (Mg, Mn, Cu, Zn, Se and Mo) quantities were estimated and a correlation analysis was carried out.

**Results:**

Results of this study show that significant levels of phenolics, flavonoids and trace metal contents were found in *Ligustrum lucidum, Paeonia suffuticosa, Salvia miltiorrhiza, Sanguisorba officinalis, Spatholobus suberectus, Tussilago farfara* and *Uncaria rhyncophylla*, which correlated well with their antioxidant and anti-inflammatory activities. Some of the plants displayed high antioxidant and anti-inflammatory activities but contained low levels of phenolics and flavonoids. Interestingly, these plants contained significant levels of trace metals (such as Zn, Mg and Se) which are likely to be responsible for their activities.

**Conclusions:**

The results indicate that the phenolics, flavonoids and trace metals play an important role in the antioxidant activities of medicinal plants. Many of the plants studied here have been identified as potential sources of new antioxidant compounds.

## Background

It is well known that reactive oxygen species (ROS), such as superoxide anion (O_2_^·-^), hydroxyl radicals (OH^·^), singlet oxygen (^1^O_2_) and hydrogen peroxide (H_2_O_2_), play a major role in the development of oxidative stress that can lead to many illnesses including cardiovascular diseases, diabetes, inflammation, degenerative diseases, cancer, anemia, and ischemia [1]. Many synthetic antioxidant agents have been developed to remediate oxidative stress. However, the factors such as high cost, lack of availability and side effects remained as major setbacks in combating oxidative stress. In this direction, natural antioxidants received a prominence as they are often free from side effects, less expensive and abundant in many plant sources [1]. Plant based antioxidant compounds [1,2] play a defensive role by preventing the generation of free radicals and hence are extremely beneficial to alleviate the diseases caused by oxidative stress [3,4]. Many investigations revealed that phenolics and flavonoids content [1,5-10] contribute to the antioxidant activities of plants. In addixtion to these classes of organic compounds, recent studies demonstrated that trace metals such as Cu, Zn, Mg, Mn and Se play a beneficial role in antioxidant mechanism [11,12].

Studies have also uncovered that phenolics and flavonoids act as excellent anti-inflammatory agents [6,13]. The anti-inflammatory properties of flavonoids have been extensively studied and beneficial effects have been demonstrated in many animal models [13]. Excess production of pro-inflammatory molecules such as TNF-α and nitric oxide (NO) are believed to be responsible for modulating inflammation besides their crucial role in immune-inflammatory response. These inflammatory molecules are also known to cause cell death and tissue damage because NO can react with the free radicals such as superoxides to produce peroxynitrite, that can lead to irreversible damage to cell membranes [14,15]. In order to search for effective natural antioxidants and anti-inflammatory compounds, current study has selected forty four traditional Chinese medicinal (TCM) plants based on their ethno-pharmacological importance.

TCM plants have history of usage in the treatment of several diseases for thousands of years [16,17]. They are used for the treatment of many ailments including cancer in China and around the world. Several compounds derived from the TCM plants are potential anticancer agents and many of them are currently in clinical trials [18]. The extracts of TCM herbs are also used as pharmaceutical and dietary supplements.

The most important aspect of natural product research is to select a rapid, easy and efficient screening method. In the current study, the antioxidant activities of selected TCM plants were evaluated using DPPH (diphenylpicrylhydrazyl) radical scavenging method and yeast based antioxidant screening assay [19]. DPPH is a stable synthetic free radical and has been widely used for measuring free radical scavenging activity [20]. The yeast based biological assay detects antioxidant activities of samples against physiologically relevant oxidants. It is believed that antioxidant activities of medicinal plants must be evaluated by more than one method (by at least two methods) in order to take into account different modes of action of a given antioxidant [21]. Hence, in this study the antioxidant and anti-inflammatory activities of selected TCM plants were measured systematically using a series of assays.

## Methods

### Plant materials

The dried plant materials were obtained from Beijing Tong Ren Tang Chinese Herbal Medicine shop, Sydney, Australia. A voucher specimen of each plant has been deposited in the laboratory. The scientific names and family names were given in Table 1. The plant materials were ground to a fine powder in a grinder before extraction.

**Table 1:** List of Chinese medicinal herbs used in this study

**S. no**	**Name of the plant species**	**Chinese name**	**Family names**	**Traditional medicinal use**	**References**
1	*Acanthopanax senticosus* Herms	NA	Araliaceae	Treatment of rheumatism, allergies and diabetics	[22]
2	*Actinidia arguta (Sieb.et Zucc.)Flarich.ex Miq.*	Teng li gen	Actinidiaceae	Anticancer and anti-allergic	[23]
3	*Akebia quinata (Houtt.)Decne.*	Ba yue zha	Lardizabalaceae	anti-phlogistic	[24]
4	*Alpinae officinarum*	Gao liang jiang	Zingaberaceae	Antioxidant	[25]
5	*Andrographis paniculata* (Burm.f.) Wall. ex Nees	Shuan xin lian	Acanthaceae	Anti-cacner.	[26]
6	*Artemisia vulgaris L.*	Ai ye	Asteraceae	Anti-cancer	[27]
7	*Asparagus cochinchinensis* (Lour.) Merr.	Tian men dong	Asparagaceae	Anti-tumour	[28]
8	*Aster tataricus* L.	Zi wan	Asteraceae	Anti-oxidant	[29]
9	*Corydalis yanhusuo* W.	Yan hu suo	Papaveraceae	Anti-cancer	[18]
10	*Curcuma zedoaria (Christm.) Roscoe*	E zhu	Zingiberaceae	Antioxidant activity, Anti-mutagenic activity and Anti-microbial activity,	[30]
11	*Cynanchum paniculatum (Bge.) Kitag*	Xu chang qing	Asclepiadaceae	Anticancer	[18]
12	*Cyperus rotundus* L.	Xiang fu	Cyperaceae	Anticancer	[18]
13	*Ducheshea indica* (Andr.) Focke.	She mei	Rosaceae	Anti-inflammatory and anti-cancer	[31]
14	*Hedyotis diffusa* Willd.	Bai hua she she cao	Rubiaceae	Antibacterial	[18]
15	*Leonurus japonicus* Houtt.	Yi mu cao	Labiatae	Anti-cancer	[32]
16	*Ligustrum lucidum Ait.*	Nv zhen zi	Moraceae	Anti-cancer	[18]
17	*Lobelia chinensis Lour.*	Ban bian lian	Campanulaceae	Antipyretic, anti-inflammatory, and antitoxic effects	[18]
18	*Lysinachia christinae* Hance.	Jin qian cao	Lysimachia	Anticancer	[18]
19	*Paeonia lactiflora* Pall.	Bai shao	Paeoniaceae	Anticancer and anti-bacterial	[33]
20	*Paeonia suffuticosa* Sndr.	Mu dan pi	Ranunculaceae	Anti-cancer	[18]
21	*Paris polyphylla* Smith	Qi ye yi zhi hua	Trilliaceae	Anti-tumour	[28]
22	*Platycodon grandiflorus* (Jacq.) A. DC.	Jie geng	Campanulaceae	Anti-cancer	[18]
23	Plantago asiatica L.	Che qian cao	Plantaginaceae	Anti-cancer	[34]
24	*Pleione bulbocadioides* (Franch.)Rolfe.	Shen ci gu	Orchidaceae	Anticancer and antibacterial	[18]
25	*Ploygala tenuifolia* Willd.	Yuan zhi	Polygalaceae	Anti- inflammatory	[35]
26	Polygonum aviculare L.	Bian cun	Polygonaceae	Anti-oxidant activity and anti- inflammatory activity	[36]
27	Poria cocos (Schw.) Wolf	Fu lin	Polyporaceae	Anti-tumuor	[28]
28	*Pogostemon cablin* Benth.	Guang huo xiang	Asteraceae	Anti-insecticidal, anti-fungal and bacteriostatic	[37]
29	*Prunella vulgaris* L.	Xia ku cao	Lamiaceae	Anticancer and anti-inflammatory properties	[38]
30	*Pseudostellaria heterophylla* (Miq.) Pax ex Pax et Hoffm.	Tai zi shen	Caryophyllaceae	Anti-tumuor	[28]
31	*Rabdosia rubescens*(Hamst.)Wuet.	Dong ling cao	Labiatae	Anti-cancer	[18]
32	*Rehmannia glutinosa* (Gaertn.) Steud.	Sheng di huang	Phrymaceae	Anti-tumour	[28]
33	*Salvia miltiorrhiza* Bunge.	Dan shen	Caspase	Anti-tumuor	[28]
34	*Sanguisorba officinalis L.*	Di yu	Rosaceae	Anti-allergic and hepatitis B virus	[18]
35	*Schizandra chinensis* (Turcz.) Baill.	Wu wei zi	Schisandraceae	Antioxidant effect	[39]
36	*Scutellaria barbata Don.*	Ban zhi lian	Labiatae	Anti-tumuor	[28]
37	*Semen coicis* L.	Yi yi ren	Gramineae	Anti-cancer	[18]
38	*Smilax glabra* Roxb.	Tu fu ling	Smilacaceae	Anti-tumuor	[28]
39	Solanum nigrum L.	Long Kui	Solanaceae	Anti-cancer	[18]
40	*Solanum lyratum Thunb.*	Bai ying	Solanaceae	Anti-tumuor	[28]
x41	*Spatholobus suberectus Dunn.*	Ji xie teng	Leguminosae	Anti-tumuor	[18]
42	*Tussilago farfara* L.	Kuan dong	Asteraceae	Anti-microbialand antioxidant	[40]
43	*Uncaria rhyncophylla* Miq	Gou Teng	Rubiaceae	Antioxidant activity and anti-inflammatory activity	[41]
44	*Viscum coloratum* (Komar.) Nakai	Hui ji sheng	Viscaceae	Anti-infammatory	[42]

NA**:**No appropriate reference is available.

### Chemicals and reagents

Gallic acid, Quercetin, 2, 2-diphenyl-1-picrylhydrazyl (DPPH), Dimethyl sulfoxide (DMSO), sodium carbonate, aluminium chloride (AlCl_3_), sodium nitrate (NaNO_2_), sodium hydroxide (NaOH), hydrogen peroxide (H_2_O_2_), Folin-Ciocalteu (F-C) reagent, ascorbic acid, 95% ethanol, bovine serum albumin (BSA), lipopolysaccharide (LPS: *E.coli* serotype 0127:B8), N-(1-1-napthyl) ethylenediamine dihydrochloride, penicillin G sodium benzyl, resazurin sodium 10%, streptomycin, sulfanilamide, tetramethyl benzidine (TMB), trypan blue were purchased from Sigma (Australia) and Lomb Scientific Pty Ltd (Australia). Antibiotics, Dulbecco’s modified eagle’s medium (DMEM), foetal bovine serum (FBS) and glutamine were purchased from GIBCO. Interferon-γ (murine) and tumor necrosis factor-α (TNF-α) – enzyme-linked immunosorbent assay (ELISA) kits were purchased from Peprotech. RAW 264.7 macrophages (ATCC number TIB-71) were obtained from American Type Culture Collection (ATCC).

### Preparation of water extracts

Approximately 3 g of each grounded plant material was autoclaved with 30 mL of deionised water at 121°C for 1 hr as described in a previous publication [6]. The extracted samples were centrifuged at 10,447 g for 20 min) and the supernatant was transferred into a 50mL volumetric flask. The residue was further rinsed two more times, pooled the extracts and the volume adjusted to 50mL. The samples were stored at −20°C until analysis.

### Preparation of ethanol extracts

Ground samples (3 g) were extracted with 30mL of 95% ethanol on water bath at 70°C for 6 hr [6]. The extracted samples were centrifuged and the supernatant was transferred into a 50 mL volumetric flask. The residue was further rinsed two more times, pooled the extracts and the volume adjusted to 50 mL with 95% ethanol. The samples were stored at −4°C until analysis. All water and ethanol extracts were filtered before analysis.

### Determination of total phenolic content

The total phenolic content was determined by Folin-Ciocalteu (F-C) colorimetric method [43]. Briefly, 50 μL of sample and 50 μL of F-C reagent were pipetted into an eppendorf tube. The contents were vortexed for 10 sec and then left at room temperature for 2 min. After 2 min, 500 μL of 5% (w/v) sodium carbonate solution was added to stop the reaction and then 400μL of distilled water was added to make up to 1mL. The vortexed reaction mixture was heated in a water bath at 45°C for 30 min and then cooled rapidly in an ice bath. Absorbance was measured at 760 nm. Gallic acid concentrations ranging from 0–300 μg/mL were prepared and the calibration curve was obtained using a linear fit (r^2^ = 0.9961). The samples were analyzed in duplicates.

### Determination of total flavonoid content

The total flavonoid content was estimated by aluminium chloride method [44]. Briefly, 0.5 mL of each sample and 300 μL of NaNO_2_ (1: 20 w/v) were pipetted into a test tube. The contents were vortexed for 10 sec and left at room temperature for 5 min. Into the mixture were then added 300 μL of AlCl_3_ (1:10 w/v), 2 mL of 1M NaOH and 1.9 mL of distilled water. After vortexing for 10 sec, the absorbance for each sample was measured at 510 nm. Quercetin concentrations ranging from 0 to 1200 μg/mL were prepared and the standard calibration curve was obtained using a linear fit (r^2^ = 0.9980). The samples were analyzed in duplicates.

### Determination of trace metal content using ICP-MS technique

The plant water extracts were digested with 5 mL of concentrated HNO_3_ (Suprapure, Merck) and 2 mL of H_2_O_2_ (Suprapure, Merck) in a clean glass beaker and heated on a hot plate and diluted to 10 mL with double deionized water (Milli-Q Millipore 18.2 MW/cm resistivity). A blank digest was also carried out in the same way. The temperature of the hot plate was maintained at around 105°C continuously for 4–5 hr in order to completely digest the sample and to sustain recovery of volatile elements. The concentration of six elements, namely Mg, Mn, Cu, Zn, Se and Mo, were measured by inductively coupled plasma-mass spectrometer (ICP-MS) (Model: Perkin Elmer® ELAN DRC II, Ontorio, Canada). The certified reference material, NIST SRM 1640a was used as a calibration standard and NIST SRM 1643e (obtained from National Institute of Standards & Technology, NIST, USA) was analyzed as an unknown along with the samples to ensure accuracy. Triplicate analysis of all the samples was conducted in order to check the precision and accuracy of the data. The overall RSD for all the samples was less than 10, and the standard error was found to be within the required analytical precision. The method has been validated according to the standard ISO guidelines.

### Free radical DPPH scavenging assay

DPPH radical scavenging assay was carried using Blois method [45]. Each plant extract (50 μL) in water and ethanol was added to a 150 μL of 62.5 μM DPPH. After 30 min of incubation, the absorbance of the reaction mixtures was measured at 492 nm using a microplate reader (Multiskan EX, Thermo Electron, USA). Ascorbate (Vitamin C), an antioxidant, was used as a positive control. A standard curve was included for each plate with a series of ascorbate concentrations (0, 10, 20, 40, 60, 80, 100, 200, 400 and 1000 μM). The free radical reduction capacity for each herbal extract was calculated as the ascorbate equivalent against the ascorbate standard curve (r^2^ = 0.9924).

### Antioxidant activity screening in a 96-well microplate high throughput assay using *Saccharomyces cerevisiae*

The antioxidant capacities of the herbal extracts were also measured using a *S. cerevisiae*-based high throughput assay [19]. *S. cerevisiae* BY4743 were cultured overnight in a 50 mL volume by inoculation of a single colony. The culture was then diluted to an optical density at 600 nm (OD_600_) of 0.2 in media, and 180 μL of each strain was added into a well in a 96-well microtitre plate where 10 μL per well of each herbal extract was also added in duplicates. 10 μL of H_2_O_2_ was added to a final concentration of 4 mM. The initial OD_600_ reading was taken using a microplate reader (Multiskan EX, Thermo Electron, USA), and the plates were then incubated in a 30°C incubator with shaking at 750 rpm. Yeast growth was monitored at reading OD_600_ at the end of 20 hours. Ascorbic acid was used as a positive control. The net growth of H_2_O_2_ induced yeast cells after the treatment of selected plant extracts was measured using the following equation

(1) $$P_{yeast\;growth}=\left(\frac{P_{\mathrm{Sample}}-P_{\mathrm{Control}}}{P_{\mathrm{Control}}}\right)\times 100$$

‘*P*_*yeast growth*_’ _=_ Net growth of H_2_O_2_ induced yeast cells after treatment with plant extracts.

‘*P*_*Sample*_’ _=_ Observed optical density of yeast cells with the treatment of plant extracts.

‘*P*_*Control*_’ _=_ Observed optical density of yeast cells with the treatment of negative control _(_H_2_O_2)._

#### Maintenance and activation of RAW 264.7 macrophages

RAW 264.7 macrophages were grown in 175 cm^2^ flasks on DMEM containing 5% FBS that was supplemented with antibiotics (1%) and glutamine (1%). The cell line was maintained in 5% CO_2_ at 37°C, with media being replaced every 3–4 days. Once cells had grown to confluence in the culture flask, they were removed using a rubber policeman, as opposed to using trypsin, which can remove membrane-bound receptors such as RAGE [6,46]. Cell suspension was concentrated by centrifuging for 3 min at 900 rpm and resuspension in a small volume of fresh DMEM (with 1% antibiotics and 5% FBS). Cell densities were estimated using a Neubauer counting chamber. Cell concentration is adjusted with DMEM (with 1% antibiotics and 5% FBS) to obtain 75,000 cells/well when 100-μL cell suspensions dispensed into the 60-inner wells of 96-well plates. Sterile distilled water was added to the outer row of wells and incubated at 37°C; 5% CO_2_ for 12 hr. From each well, conditioned medium was replaced with fresh serum-free medium. For assays with extracts, 50-μL volume of the dilutions (in water) was added an hour prior to addition of activator. Due to the often inconsistent nature of LPS at activating cells, a combination of 25 μg/mL LPS and 10 U/ml IFN-γ, both in DMEM, was used for activation. Usually a maximum dose of the extracts used was 2.5 mg/mL and a minimum of 6 doses made by serial dilution. Then the cells were incubated for 24 hr at 37°C and 5% CO_2_. Cells with media alone were used as negative control and activated cells as positive control.

#### Determination of nitric oxide production by Griess assay

Nitric oxide is determined by Griess reagent quantification of nitrite; one of its stable reaction products. Griess reagent is freshly made up of equal volumes of 1% sulphanilamide and 0.1% napthyethylene-diamine in 5% HCl. In the presence of nitrite this reagent forms a violet colour. From each well 70 μL of supernatant was transferred to a fresh 96-well plate and mixed with 70 μL of Griess reagent and the colour produced was measured at 540 nm. The remaining supernatant that was removed from each well was used for TNF-α assay using a commercial sandwich ELISA.

#### Determination of cell viability by Alamar Blue assay

Alamar Blue assay is a calorimetric assay involving the cellular reduction of resazurin to resorufin. 100 μL of Alamar Blue solution (10% Alamar Blue (Resazurin) in DMEM media) was added to each well and incubated at 37°C for 1–2 hr. Fluorescence was measured (excitation @ 545 nm and emission @ 595 nm) and expressed as a percentage of that in control cells after background fluorescence was subtracted.

#### TNF-α determination by ELISA

Sandwich ELISA was used according to the manufacturer's manual (Peprotech) to determine TNF-α concentration. Capture antibody was used at a concentration of 0.5 μg/mL in PBS (1.9 mM NaH_2_PO_4_, 8.1 mM Na_2_HPO_4_, 154 mM NaCl; pH 7.4). Serial dilutions of TNF-α standard from 0 to 1000 pg/mL in diluent (0.05% Tween-20, 0.1% BSA in PBS) were used as internal standard. TNF-α was detected with a biotinylated second antibody and an avidin peroxidase conjugate with TMB as detection reagent. The color development was monitored at 655 nm, taking readings after every 5 min. After 25 min the reaction was stopped using 0.5 M sulphuric acid and the absorbance was measured at 450 nm.

#### Data presentation and analysis

As the experiments were done in duplicates, the results were expressed in mean ± standard deviation. In addition, linear relationships and significance tests of these data sets were also conducted. GraphPad prism 5.01 was used for growth curve analysis in dose-dependent experiments and to determine the IC_50_ values for NO and TNF-α inhibition.

## Results and discussion

### Total phenolics and flavonoids content in the selected plants

The total phenolics and flavonoids content of selected 44 herbal extracts were measured using F-C reagent and aluminium chloride methods respectively. These results obtained for water and ethanol extracts of the plants are presented in Table 2.

**Table 2:** The total phenolics and flavonoids content together with DPPH free radical scavenging activities of ethanol and water extracts of plant material, and the antioxidant activity against yeast oxidative stress

	**DPPH scavenging activity (μM Ascorbate equivalent / g)**^**1**^			**Total phenolics content (GAE mg/g)**^**3**^		**Total flavonoids content (QE mg/g)**^**3**^	
**Name of the plants**	**Water extracts**	**Ethanol Extracs**	**% inhibition of Yeast oxidation of water extracts**^**2**^	**Water extracts**	**Ethanol extracts**	**Water extracts**	**Ethanol extracts**
*Acanthopanax senticosus*	186.9 ± 0.71	99.93 ± 1.01	10.11	8.52 ± 3.00	3.89 ± 0.00	24.21 ± 0.98	14.37 ± 0.00
*Actinidia arguta*	183.4 ±1.41	104.21 ± 0	7.24	4.71 ± 0.55	15.26 ± 0.44	13.99 ± 0.98	62.85 ± 0.98
*Akebia quinata*	195.4 ±1.41	99.93 ± 4.04	14.76	7.09 ± 4.64	8.22 ± 0.44	16.66 ± 1.96	41.4 ± 5.87
*Alpinae officinarum*	201.9 ± 0.71	105.29 ± 0.51	0	11.78 ± 0.00	23.35 ± 3.14	26.81 ± 0.98	87.39 ± 0.98
*Andrographis paniculata*	171.4 ± 1.41	76.00 ± 4.55	13.33	5.09 ± 0.41	1.92 ± 1.03	8.94 ± 0.98	7.22 ± 1.96
*Artemisia vulgaris*	181.9 ±0.71	98.14 ± 1.52	5.32	24.91 ± 0.27	7.43 ± 1.44	79.68 ± 1	16.43 ± 3.91
*Asparagus cochinchinensis*	55.9 ± 12.02	5.64 ± 1.01	16.37	2.67 ± 0.96	0.91 ± 3.01	1.71 ± 0.98	1.08 ± 0.98
*Aster tataricus*	119.4 ± 2.83	49.93 ± 2.02	0	15.17 ± 0.27	6.95 ± 0.00	39.82 ± 0.98	22.79 ± 0.98
*Corydalis yanhusuo*	39.9 ± 0.71	42.43 ± 5.56	14.81	3.51 ± 1.36	1.28 ± 1.48	1.57 ± 0.96	2.11 ± 0.98
*Curcuma zedoaria*	158.9 ±0.71	108.14 ± 1.52	10.98	6.14 ± 0.55	7.38 ± 0	11.67 ± 0.98	25.38 ± 2.93
*Cynanchum paniculatum*	138.9 ±0.71	32.79 ± 1.01	6.13	6.31 ± 0	3.05 ± 0.89	3.3 ± 0	10.03 ± 0.00
*Cyperus rotundus*	114.9 ± 4.95	85.29 ± 2.53	5.06	3.46 ± 0.82	1.89 ± 0.44	5.01 ± 0.98	17.97 ± 0.98
*Ducheshea indica*	202.4 ± 1.41	102.07 ± 4.04	2.34	28.62 ± 0.68	11.8 ± 0.44	22.31 ± 4.89	15.25 ± 1.96
*Hedyotis diffusa*	160.9 ± 6.36	42.79 ± 2.02	8.54	5.17 ± 0.27	2.25 ± 0.00	7.37 ± 1.96	8.5 ± 0.98
*Leonurus japonicus*	159.9 ± 0.71	41.36 ± 4.04	1.93	6.46 ± 0.82	0.84 ± 2.66	8.22 ± 1.96	5.39 ± 1.96
*Ligustrum lucidum*	194.9 ±0.71	107.79 ± 0	12.02	29.1 ± 3.28	19 ± 0.78	108.42 ± 1.96	24.88 ± 0.98
*Lobelia chinensis*	101.4 ±0	12.79 ± 2.02	−1.94	4.59 ± 0.14	2.74 ± 0.44	2.79 ± 0.98	8.92 ± 3.91
*Lysinachia christinae*	193.9 ± 3.54	66.00 ± 0.51	0	4.24 ± 0.55	3.91 ± 0.30	2.63 ± 1.96	10.29 ± 0.00
*Paeonia lactiflora*	172.9 ± 3.54	113.14 ± 0.51	16.46	7.65 ± 0.41	6.3 ± 0.44	3.39 ± 1.96	6.42 ± 0.98
*Paeonia suffuticosa*	200.4 ± 0.00	111.71 ± 0.51	15.16	19.96 ± 1.09	19.74 ± 0.39	25.85 ± 1.96	31.40 ± 2.93
*Paris polyphylla*	19.9 ± 2.12	45.29 ± 1.52	0	1.23 ± 1.36	1.01 ± 3.69	0.72 ± 1.96	2.60 ± 1.96
*Platycodon grandiflorus*	30.9 ± 3.54	14.93 ± 4.04	21.77	4.58 ± 1.50	0.79 ± 0.26	2.93 ± 2.93	1.52 ± 0.96
*Plantago asiatica*	184.4 ±9.9	49.93 ± 1.01	19.77	6.7 ± 0.96	0.82 ± 0.39	15.57 ± 3.91	3.32 ± 1.96
*Pleione bulbocadioides*	27.4 ± 7.07	85.64 ± 1.01	0	1.62 ± 0.27	1.05 ± 2.51	2.09 ± 0.98	1.73 ± 0.98
*Ploygala tenuifolia*	109.4 ± 2.83	53.14 ± 2.53	3.23	12.01 ± 0.00	12.15 ± 0.44	9.3 ± 3.91	37.55 ± 3.91
*Polygonum aviculare*	201.4 ±2.83	88.86 ± 1.52	3.67	5.9 ± 0.82	11.72 ± 0.15	9.15 ± 0.00	14.15 ± 0.00
*Poria cocos*	28.9 ±3.54	109.57 ± 0.51	12.11	0.45 ± 0	1.43 ± 3.53	0	0.68 ± 0.98
*Pogostemon cablin*	103.4 ± 4.24	26.00 ± 1.52	12.07	4.23 ± 0.68	1.88 ± 0.13	5.54 ± 0.98	4.08 ± 0.98
*Prunella vulgaris*	106.9 ±20.51	106.36 ± 0.00	0	16.02 ± 0.27	1.66 ± 0.74	20.27 ± 3.91	15.62 ± 1.96
*Pseudostellaria heterophylla*	20.9 ±12.02	32.43 ± 4.55	36.13	1.74 ± 0.82	1.58 ± 1.83	0.59 ± 0.00	1.94 ± 0.98
*Rabdosia rubescens*	144.4 ± 2.83	13.50 ± 4.04	8.63	17.47 ± 0.55	2.41 ± 2.96	40.66 ± 1.96	8.35 ± 2.93
*Rehmannia glutinosa*	34.4 ± 0.00	20.29 ± 0.51	17.94	5.99 ± 1.23	0.88 ± 0.13	6.12 ± 0.98	2.07 ± 1.96
*Salvia miltiorrhiza*	202.4 ± 4.24	104.93 ± 0.00	12.72	66.27 ± 0.14	4.97 ± 0.15	133.93 ± 0.98	27.05 ± 2.93
*Sanguisorba officinalis*	191.9 ±0.71	64.21 ± 2.02	−5.47	148.09 ± 2.46	121.42 ± 0.78	129.53 ± 0.98	213.23 ± 0
*Schizandra chinensis*	73.9 ± 3.54	33.86 ± 1.52	4.8	7.95 ± 2.73	11.91 ± 0.15	11.36 ± 7.82	31.3 ± 1.96
*Scutellaria barbata*	188.4 ±1.41	93.5 ± 1.01	10.11	14.97 ± 2.73	9.26 ± 0.15	67.41 ± 3.91	20.62 ± 0.98
*Semen coicis*	−100.1 ± 0.71	11.00 ± 3.54	12.37	0.34 ± 2.32	1.39 ± 0.65	0.83 ± 1.96	16.05 ± 2.93
*Smilax glabra*	135.9 ± 2.12	109.21 ± 0.00	5.84	6.19 ± 2.73	12.62 ± 0.89	45.28 ± 1.96	45.95 ± 4.89
*Solanum nigrum*	190.4 ±0	50.64 ± 3.03	5.17	4.2 ± 0.27	2.98 ± 2.48	6.43 ± 0.98	4.41 ± 4.89
*Solanum lyratum*	192.4 ±1.41	47.07 ± 1.01	1.03	3.63 ± 0.41	2.55 ± 1.77	2.62 ± 2.93	5.18 ± 0.98
*Spatholobus suberectus*	184.4 ±0	98.14 ± 2.53	10.63	24.11 ± 0.96	34.02 ± 4.05	165.16 ± 1.96	69.19 ± 0.00
*Tussilago farfara*	198.9 ± 0.71	113.5 ± 0.00	20.9	25.72 ± 2.87	9.68 ± 0.15	65.17 ± 0.98	41.67 ± 0.98
*Uncaria rhyncophylla*	181.4 ± 2.83	98.50 ± 1.01	13.24	32.38 ± 0.55	6.75 ± 0.30	58.91 ± 0.00	22.13 ± 1.96
*Viscum coloratum*	129.9 ±12.02	57.07 ± 0.00	23.69	3.34 ± 3.00	9.06 ± 0.00	3.07 ± 0.98	28.58 ± 3.91

^1^DPPH free radical scavenging activity was measured in terms of equivalent of ascorbate (μM).

^2^Yeast oxidative stress was measured on the basis of survival of yeast cells (yeast growth) after treatment with H_2_O_2_.

^3^Total phenolics and flavonoid contents were expressed in gallic acid equivalent (GAE mg/g) and quercetin equivalent (QE mg/g) respectively.

### Water extracts

As can be seen from Table 2, significant phenolics content was observed in water extracts of *S. officinalis* (148.09 GAE mg/g)*, S. miltiorrhiza* (66.27 GAE mg/g) and moderate levels in *U. rhyncophylla* (32.38 GAE mg/g), *L. lucidum* (29.1 GAE mg/g), *Ducheshea indica* (28.62 GAE mg/g), *T. farfara* (25.72 GAE mg/g), *Artemisia vulgaris* (24.91 GAE mg/g) and *S. suberectus* (24.11 GAE mg/g). High levels of flavonoids content was found in water extracts of *S. suberectus* (165.16 QE mg/g), *S. miltiorrhiza* (133.93 QE mg/g), *S. officinalis* (129.53 QE mg/g), *L. lucidum* (108.42 QE mg/g) and moderate to low levels in *Artemisia vulgaris* (79.68 QE mg/g), *T. farfara* (65.17 QE mg/g), *U. rhyncophylla* (58.91 QE mg/g), *Rabdosia rubescens* (40.66 QE mg/g), *Aster tataricus* (39.82 QE mg/g), *Alpinae officinarum* (26.81 QE mg/g), *Acanthopanaxsenticosus* (24.21 QE mg/g) and D. *indica* (22.31 QE mg/g).

### Ethanol extracts

Amongst all the ethanol extracts, high levels of phenolics content was found in *S. officinalis* (121.42 GAE mg/g) and moderate levels in *S. suberectus* (34.02 GAE mg/g) and *A. officinarum* (23.35 GAE mg/g) (Table 2). High flavonoid content was found in ethanol extracts of *S. officinalis* (213.23 QE mg/g), *A. officinarum* (87.39 QE mg/g) and moderate to low levels in *S. suberectus* (69.10 QE mg/g), *Actinidia arguta* (62.85 QE mg/g), *S. glabra* (45.95 QE mg/g), *T. farfara* (41.67 QE mg/g), *Ploygala tenuifolia* (37.55 QE mg/g), *Paeonia suffuticosa* (31.4 QE mg/g) and *Viscum coloratum* (28.58 QE mg/g).

It is interesting to note that *A. officinarum, S. suberectus, S. officinalis, T. farfara, S. miltiorrhiza* and *U. rhyncophylla* have been found to have significant levels of phenolics and flavonoids content in both water and ethanol extracts. A close observation of the results presented in this paper indicates that the total phenolics and flavonoids content varied amongst the selected plant species and in different extracts. These variations of antioxidant contents have been discussed in terms of the observed bioactivities of the plants.

### Trace metal content in the selected plants

In the current study, trace metal content (Mg, Mn, Cu, Zn, Se and Mo) of water extracts was determined using ICP-MS technique (Table 3). Among all the metals, Mn was the most abundant while Se and Mo were least abundant. Significant quantity of Cu was found in *Poria cocos* (230.2 μg/g), *A. arguta* (201.75 μg/g). High levels of Zn was found in *P. cocos* (1841.43 μg/g), *V. coloratum* (977.52 ÎÂ¼g/g), *A. arguta* (969.13 ÎÂ¼g/g) and *S. officinalis* (909.37 μg/g). Of all the analyzed trace metals, Mn was highly abundant in *Curcuma zedoaria* (2312.15 μg/g), *U. rhyncophylla* (2770.85 μg/g), *A. officinarum* (2212.53 μg/g), *Hedyotis diffusa* (1778.79 μg/g), *P. cocos* (1257.65 μg/g) and *S. suberectus* (1211.85 μg/g). The Mg content amongst all the plants was relatively high (expressed in mg/g in Table 3), while Selenium and Molybdenum were least abundant.

**Table 3:** The trace metal content of water extracts of selected medicinal plants

**Name of the plants**	Cu (μg/g)^*^	Zn (μg/g)^*^	Mn (μg/g)^*^	**Mg (mg/g)**^**#**^	Se (μg/g)^*^	Mo (μg/g)^*^
*Acanthopanax senticosus*	30.86 ± 0.61	183.33 ± 3.91	35.01 ± 0.86	10.35 ± 0.41	43.04 ± 1.41	0.73 ± 0.03
*Actinidia arguta*	201.75 ± 1.28	969.13 ± 6.68	182.96 ± 1.46	38.44 ± 0.49	5.9 ± 0.06	2.96 ± 0.04
*Akebia quinata*	29.28 ± 1.72	310.65 ± 19.77	93.36 ± 6.85	7.24 ± 0.85	1.74 ± 0.17	0.12 ± 0.02
*Alpinae officinarum*	19.94 ± 0.73	276.02 ± 10.99	2212.53 ± 101.6	10.56 ± 0.78	1.92 ± 0.12	0.22 ± 0.02
*Andrographis paniculata*	61.13 ± 1.77	245.62 ± 7.69	542.3 ± 19.6	65.89 ± 3.81	13.93 ± 0.67	0.78 ± 0.05
*Artemisia vulgaris*	43.8 ± 1.27	135.75 ± 4.28	321.4 ± 11.69	18.6 ± 1.08	16.28 ± 0.79	0.55 ± 0.03
*Asparagus cochinchinensis*	6.71 ± 0.75	77.24 ± 9.37	54.5 ± 7.63	3.05 ± 0.68	1.86 ± 0.35	0.13 ± 0.03
*Aster tataricus*	3.47 ± 0.26	30.35 ± 2.48	46.6 ± 4.39	2.78 ± 0.42	1.44 ± 0.18	0.09 ± 0.01
*Corydalis yanhusuo*	7.73 ± 0.61	154.73 ± 13.17	42.99 ± 4.28	9.61 ± 1.51	0.48 ± 0.06	0.87 ± 0.14
*Curcuma zedoaria*	32.74 ± 0.5	45.85 ± 0.75	2312.15 ± 43.84	29.26 ± 0.89	13.7 ± 0.35	3.27 ± 0.1
*Cynanchum paniculatum*	23.57 ± 0.78	171.76 ± 6.16	53.96 ± 2.23	12.39 ± 0.82	2.92 ± 0.16	0.51 ± 0.04
*Cyperus rotundus*	21.03 ± 0.61	79.54 ± 2.51	27.35 ± 0.99	6.41 ± 0.37	0.54 ± 0.03	0.39 ± 0.02
*Ducheshea indica*	18.55 ± 0.88	175.82 ± 9.06	156.07 ± 9.28	19.58 ± 1.86	2.72 ± 0.22	0.37 ± 0.04
*Hedyotis diffusa*	59.23 ± 1.06	304.91 ± 5.89	1778.79 ± 39.63	59.31 ± 2.11	42.38 ± 1.26	3 ± 0.11
*Leonurus japonicus*	82.62 ± 1.53	848.36 ± 17.01	214.72 ± 4.97	40.24 ± 1.49	47.03 ± 1.45	2.57 ± 0.11
*Ligustrum lucidum*	16.51 ± 0.86	250.49 ± 14.17	72.87 ± 4.76	9.43 ± 0.99	0.4 ± 0.03	1.41 ± 0.15
*Lobelia chinensis*	9.37 ± 0.75	236.66 ± 20.53	124.26 ± 12.44	2.26 ± 0.36	0.64 ± 0.09	1.2 ± 0.2
*Lysinachia christinae*	68.01 ± 1.31	461.07 ± 9.61	22.96 ± 0.55	16.46 ± 0.63	27.88 ± 0.89	1.12 ± 0.04
*Paeonia lactiflora*	2.89 ± 0.12	130.61 ± 5.95	147.3 ± 7.75	9.73 ± 0.82	−0.11 ± −0.01	0.13 ± 0.01
*Paeonia suffuticosa*	2.42 ± 0.10	59.72 ± 2.81	29.38 ± 1.59	4.1 ± 0.36	0.57 ± 0.04	0.07 ± 0.01
*Paris polyphylla*	NA	NA	NA	NA	NA	NA
*Platycodon grandiflorus*	12.73 ± 0.96	180.17 ± 14.78	56.28 ± 5.33	12.41 ± 1.88	0.83 ± 0.11	1.39 ± 0.22
*Plantago asiatica*	59.58 ± 1.23	298.09 ± 6.68	238.28 ± 6.16	8.67 ± 0.36	14.63 ± 0.5	1.29 ± 0.06
*Pleione bulbocadioides*	NA	NA	NA	NA	NA	NA
*Ploygala tenuifolia*	21.61 ± 1.31	43.25 ± 2.84	47.82 ± 3.62	5.53 ± 0.67	0.65 ± 0.07	0.2 ± 0.03
*Polygonum aviculare*	34.48 ± 0.98	294.6 ± 9.09	5.89 ± 0.21	36.32 ± 2.07	9.81 ± 0.47	0.77 ± 0.05
*Poria cocos*	230.2 ± 1.16	1841.43 ± 10.09	1257.65 ± 7.95	7.53 ± 0.08	0.52 ± 0	3.75 ± 0.04
*Pogostemon cablin*	64.71 ± 2.11	565.31 ± 17.51	392.13 ± 13.76	83.3 ± 4.26	5.81 ± 0.27	4.66 ± 0.37
*Prunella vulgaris*	9.76 ± 0.26	128.12 ± 3.7	522.57 ± 17.4	14.98 ± 0.8	17.68 ± 0.79	0.45 ± 0.03
*Pseudostellaria heterophylla*	3.53 ± 0.18	274.37 ± 15.37	270.58 ± 17.48	4.19 ± 0.43	1.1 ± 0.10	0.36 ± 0.04
*Rabdosia rubescens*	52.69 ± 1.25	284.29 ± 7.30	692.42 ± 20.5	62.03 ± 2.94	8.54 ± 0.34	5.21 ± 0.26
*Rehmannia glutinosa*	6.86 ± 0.82	110.9 ± 14.37	6.61 ± 0.99	1.46 ± 0.35	0.55 ± 0.11	0.08 ± 0.02
*Salvia miltiorrhiza*	14.45 ± 1.12	66.17 ± 5.54	76.18 ± 7.35	23.83 ± 3.68	0.51 ± 0.07	0.13 ± 0.02
*Sanguisorba officinalis*	74.89 ± 1.09	909.37 ± 14.34	274.25 ± 4.99	25.66 ± 0.75	4.06 ± 0.1	1.2 ± 0.04
*Schizandra chinensis*	5.27 ± 0.35	168.34 ± 12.17	543.02 ± 45.31	13.4 ± 1.79	0.35 ± 0.04	0.54 ± 0.07
*Scutellaria barbata*	45.27 ± 0.56	290.33 ± 3.92	634.07 ± 9.87	58.94 ± 1.47	3.08 ± 0.06	1.11 ± 0.03
*Semen coicis*	91.28 ± 1.07	367.05 ± 4.65	367.84 ± 5.38	28.46 ± 0.67	5 ± 0.11	6.97 ± 0.17
*Smilax glabra*	NA	NA	NA	NA	NA	NA
*Solanum nigrum*	71.07 ± 2.01	404.88 ± 12.4	244.61 ± 8.64	57.18 ± 3.23	4.83 ± 0.23	4.46 ± 0.26
*Solanum lyratum*	74.33 ± 1.86	596.51 ± 16.14	9.22 ± 0.29	11.98 ± 0.6	0.89 ± 0.04	0.45 ± 0.02
*Spatholobus suberectus*	27.63 ± 0.56	188.26 ± 4.1	1211.85 ± 30.48	3.27 ± 0.13	16.65 ± 0.56	0.41 ± 0.02
*Tussilago farfara*	10.16 ± 0.84	96.79 ± 8.71	17.52 ± 1.82	2.13 ± 0.35	1.87 ± 0.26	0.18 ± 0.03
*Uncaria rhyncophylla*	8.25 ± 0.25	400.03 ± 12.95	2770.85 ± 103.53	7.91 ± 0.47	17.28 ± 0.86	0.19 ± 0.01
*Viscum coloratum*	24.77 ± 0.97	977.52 ± 41.43	392.4 ± 19.19	16.65 ± 1.3	4 ± 0.26	0.4 ± 0.03

NA: not applicable.

^*^ Trace metal contents are expressed in μg/g of dry plant samples.

^#^ Mg content was measured in mg/g.

### Antioxidant activities

The antioxidant activities of 44 selected medicinal herbs were evaluated by two methods, namely, DPPH free radical scavenging and yeast based antioxidant screening assay [19,47,48].

The water extracts of *S. nigrum, L. lucidum, Polygonum aviculare, S. lyratum, Akebia quinata, S. officinalis, P. suffuticosa, S. miltiorrhiza, A. officinarum, Lysinachia christinae, D. indica* and *T. farfara* shown significantly high free radical scavenging ability which were more than 190 μM ascorbate equiv/g (Table 2). In ethanol extracts, significant DPPH scavenging activity was found in *T. farfara*, *Paeonia lactiflora, P. suffuticosa*, *P. cocos*, *S. glabra, C. zedoaria, L. lucidum, Prunella vulgaris, A. officinarum*, *S. miltiorrhiza*, and *A. arguta, and D. indica* which were in the range of 98.14 – 113.5 μM ascorbate equiv/g (Table 2). It is interesting to note that the water extracts have displayed more scavenging activity than the ethanol extracts.

Antioxidant activities of water extracts of the selected plants were also evaluated based on their ability to inhibit the H_2_O_2_ induced yeast oxidative stress (Table 2). These results revealed that the plants *Pseudostellaria heterophylla* (36.13%), *V. coloratum* (23.69%), *Platycodon grandiflorus* (21.77%), *T. farfara* (20.9%), *Plantago asiatica* (19.77%) and *Rehmannia glutinosa* (17.94%) showed high antioxidant activity. It can be noted from Table 2 that, *T. farfara, L. lucidum, P. suffuticosa, S. miltiorrhiza, P. lactiflora* and *A. senticosus* have displayed significant antioxidant activity in both DPPH method and yeast model.

### Anti-inflammatory activities of plant extracts

The anti-inflammatory properties of water extracts of the selected medicinal herbs were evaluated on the basis of their ability to inhibit the production of NO and TNF-α in LPS and IFN-γ activated mouse macrophages. Toxicity of the plant extracts was determined using the Alamar Blue assay.

As can be seen from these results (Table 4), the extracts of *A. vulgaris, A. arguta, S. officinalis, S. suberectus, S. barbata, P. asiatica*, *Pogostemonss cablin, P. suffuticosa, H. diffusa, L. japonicus, A. paniculata, L. christinae, D. indica, U. rhyncophylla* and *R. rubescens* have down regulated NO production with IC_50_ values of less than 0.1 mg/mL without significantly affecting the cell viability (> 80). Results (Table 4) also revealed that the inhibition efficiency of plant extracts with respect to NO production was superior when compared to that of TNF-α production. Amongst all the plants, *S. officinalis* (IC_50 =_ 0.07 mg/mL), *P. asiatica* (IC_50 =_ 0.1 mg/mL) and *A. paniculata* (IC_50 =_ 0.1 mg/mL) have displayed greater inhibition of TNF-α production as well as significant down regulation of NO production and showed less toxicity.

**Table 4:** Anti-inflammatory activities of water extracts of the selected plants

**Name of the plants**	**IC**_**50**_**for the inhibition of NO production (mg/ml)**	**Cell viability (% of cell survival)**^&^	**IC**_**50**_**for the inhibition of TNF-α production (mg/ml)**	**Cell viability (% of cell survival)**^&^
*Acanthopanax senticosus*	0.12 ± 0.08	93.85 ± 8.7	1.23 ± 0	106.80 ± 11.6
*Actinidia arguta*	0.07 ± 0.01	90.3 ± 1.6	0.73 ± 0.25	83.05 ± 4.6
*Akebia quinata*	0.43 ± 0.05	105.5 ± 7.8	1.63 ± 0.4	84.5 ± 19
*Alpinae officinarum*	0.12 ± 0.01	92.80 ± 5.1	0.50 ± 0	85.55 ± 13.4
*Andrographis paniculata*	0.05 ± 0.01	85.20 ± 6.6	0.10 ± 0	77.60 ± 7.2
*Artemisia vulgaris*	0.05 ± 0	93.2 ± 6.6	0.21 ± 0.03	70.7 ± 0.6
*Asparagus cochinchinensis*	1.49 ± 1.04	98.35 ± 3.7	>2.5	NA
*Aster tataricus*	0.14 ± 0.08	98.95 ± 1.5	2.30 ± 0.09	99.70 ± 0.5
*Corydalis yanhusuo*	0.10 ± 0.01	70.70 ± 9.8	0.92 ± 0.05	62.70 ± 1.6
*Curcuma zedoaria*	0.25 ± 0.02	81.1 ± 8	1.79 ± 0.23	72.2 ± 1.5
*Cynanchum paniculatum*	0.24 ± 0.29	96.5 ± 1.1	NA^#^	NA
*Cyperus rotundus*	0.35 ± 0.37	86.60 ± 19	2.39 ± 0.64	107.50 ± 10.6
*Ducheshea indica*	0.04 ± 0.02	100.6 ± 1.2	0.38 ± 0	73.95 ± 2.1
*Hedyotis diffusa*	0.05 ± 0.00	85.60 ± 0	0.70 ± 0.08	57.30 ± 3.1
*Leonurus japonicus*	0.04 ± 0.00	78.35 ± 6.2	0.18 ± 0.11	62.00 ± 2.5
*Ligustrum lucidum*	0.14 ± 0.13	88.8 ± 15.8	0.3 ± 0.11	85.6 ± 20.4
*Lobelia chinensis*	0.36 ± 0.32	91.5 ± 13.5	1.67	51.3 ± 68.9
*Lysinachia christinae*	0.06 ± 0.00	88.50 ± 0	0.85 ± 0.14	68.90 ± 3.1
*Paeonia lactiflora*	0.30 ± 0.02	92.80 ± 1	1.07 ± 0.11	72.15 ± 0.5
*Paeonia suffuticosa*	0.09 ± 0.00	90.30 ± 5.7	0.64 ± 0.09	63.10 ± 3.1
*Paris polyphylla*	0.13 ± 0.06	91.35 ± 6.2	0.30 ± 0.40	67.1 ± 1.6
*Platycodon grandiflorus*	0.43 ± 0.08	83.05 ± 3.6	1.79 ± 0.11	59.45 ± 2.1
*Plantago asiatica*	0.05 ± 0.00	81.9 ± 0	0.1	70 ± 8.7
*Pleione bulbocadioides*	0.58 ± 0.39	101.5 ± 0.7	1.59 ± 0.22	98.6 ± 2.0
*Ploygala tenuifolia*	0.10 ± 0.08	89.55 ± 13.8	0.67 ± 0.05	52.55 ± 12.8
*Polygonum aviculare*	0.23 ± 0.26	87.0 ± 9.20	0	NA
*Poria cocos*	0.34 ± 0.01	85.9 ± 9.8	0	NA
*Pogostemon cablin*	0.07 ± 0.00	90.65 ± 9.3	0.42 ± 0.22	67.80 ± 0.6
*Prunella vulgaris*	0.12 ± 0.11	93.90 ± 6.6	2.38 ± 0.4	94.20 ± 4
*Pseudostellaria heterophylla*	1.06 ± 0.41	109.0 ± 12.7	>2.5	NA
*Rabdosia rubescens*	0.05 ± 0.01	90.65 ± 2.1	0.36 ± 0.08	65.60 ± 2.5
*Rehmannia glutinosa*	1.05 ± 0.92	91.70 ± 11.7	>2.5	NA
*Salvia miltiorrhiza*	0.20 ± 0.05	95.35 ± 4.6	0.90 ± 0.06	62.00 ± 7.6
*Sanguisorba officinalis*	0.03 ± 0.01	84.2 ± 5.2	0.07 ± 0.01	72.6 ± 2.1
*Schizandra chinensis*	0.27 ± 0.32	88.05 ± 16.9	2.31 ± 0.09	107.65 ± 11.8
*Scutellaria barbata*	0.05 ± 0.03	98.2 ± 2.5	0.6	80.5 ± 20.5
*Semen coicis*	0.63 ± 0.03	95.40 ± 5.7	1.17 ± 0.22	94.60 ± 5.7
*Smilax glabra*	0.36 ± 0.38	92.7 ± 13.2	2.35 ± 0.22	104.5 ± 6.4
*Solanum nigrum*	0	NA	1.24 ± 0.2	67.5 ± 2.1
*Solanum lyratum*	0.49 ± 0.02	107.5 ± 10.6	1.68 ± 0.89	75.4 ± 7.2
*Spatholobus suberectus*	0.06 ± 0.02	96.8 ± 1.6	0.2 ± 0.04	82.3 ± 15.8
*Tussilago farfara*	0.33 ± 0.24	101.0 ± 1.4	>2.5	NA
*Uncaria rhyncophylla*	0.05 ± 0.02	98.80 ± 1.7	0.45 ± 0.05	92.90 ± 11.5
*Viscum coloratum*	0.43 ± 0.16	89.20 ± 8.2	2.46 ± 0	104.50 ± 6.4

NA: Not analyzed.

^&^Cell viability was measured at appropriate IC_50_ values corresponding to the inhibition of NO and TNF-α.

**Figure 1:**
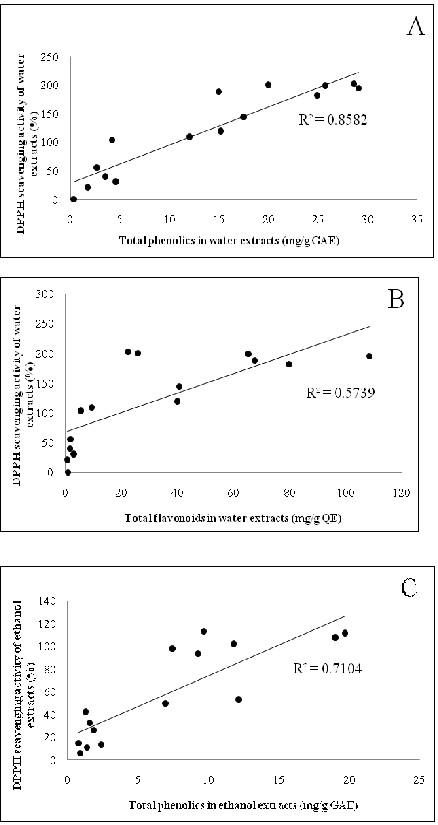
**Correlation between DPPH free radical scavenging activity and the total phenolics content in: (A) water extracts and (C) ethanol extracts.** Correlation between DPPH free radical scavenging activity and total flavonoid content in: (**B**) water extracts and (**D**) ethanol extracts.

In order to understand the relationship between the antioxidant activities and polyphenolic content (total phenolics and flavonoids), the selected 44 herbs have been classified into two groups based on the correlation between antioxidant activity and polyphenol content. The first group (consisting of fifteen plants) displayed good relationship between total phenolics / flavonoids content and antioxidant activities (Table 5 and Figure 1). The remaining plants are classified as the second group which did not show a clear correlation between the antioxidant activity and their polyphenol content. The correlation between the DPPH scavenging activity and the total phenolics and flavonoids content of water extracts of the first group of fifteen plants was found to be highly significant (Figure 1A and 1B). Similar correlation was also observed for their ethanol extracts (Figure 1C and 1D). The correlations observed in this study, for the first group of plants, are in good agreement with the literature reports that the polyphenolics are the major antioxidant compounds in medicinal plants [1,6-10,47-50].

**Table 5:** **The total phenolics and flavonoids content together with the antioxidant activity of fifteen medicinal plants (first group of plants)**^**#**^

		**DPPH scavenging activity (μM ascorbate equiv/g)**^**1**^			**Phenolics results (GAE mg/g)**^**3**^		**Flavonoids results (QE mg/g)**^**3**^	
**S. No**	**Name of the plants**	**Water extracts**	**Ethanol extracts**	**% inhibition of Yeast oxidation of water extracts**^**2**^	**Water extracts**	**Ethanol extracts**	**Water extracts**	**Ethanol extracts**
1	*Semen coicis*	−100.1 ± 0.71	11.00 ± 3.54	12.37	0.34 ± 2.32	1.39 ± 0.65	0.83 ± 1.96	16.05 ± 2.93
2	*Pogostemon cablin*	103.4 ± 4.24	26.00 ± 1.52	12.07	4.23 ± 0.68	1.88 ± 0.13	5.54 ± 0.98	4.08 ± 0.98
3	*Pseudostellaria heterophylla*	20.9 ±12.02	32.43 ± 4.55	36.13	1.74 ± 0.82	1.58 ± 1.83	0.59 ± 0.00	1.94 ± 0.98
4	*Paeonia suffuticosa*	200.4 ± 0.00	111.71 ± 0.51	15.16	19.96 ± 1.09	19.74 ± 0.39	25.85 ± 1.96	31.40 ± 2.93
5	*Corydalis yanhusuo*	39.9 ± 0.71	42.43 ± 5.56	14.81	3.51 ± 1.36	1.28 ± 1.48	1.57 ± 0.96	2.11 ± 0.98
6	*Ploygala tenuifolia*	109.4 ± 2.83	53.14 ± 2.53	3.23	12.01 ± 0.00	12.15 ± 0.44	9.3 ± 3.91	37.55 ± 3.91
7	*Ducheshea indica*	202.4 ± 1.41	102.07 ± 4.04	2.34	28.62 ± 0.68	11.8 ± 0.44	22.31 ± 4.89	15.25 ± 1.96
8	*Asparagus cochinchinensis*	55.9 ± 12.02	5.64 ± 1.01	16.37	2.67 ± 0.96	0.91 ± 3.01	1.71 ± 0.98	1.08 ± 0.98
9	*Tussilago farfara*	198.9 ± 0.71	113.5 ± 0.00	20.9	25.72 ± 2.87	9.68 ± 0.15	65.17 ± 0.98	41.67 ± 0.98
10	*Aster tataricus*	119.4 ± 2.83	49.93 ± 2.02	0	15.17 ± 0.27	6.95 ± 0.00	39.82 ± 0.98	22.79 ± 0.98
11	*Rabdosia rubescens*	144.4 ± 2.83	13.50 ± 4.04	8.63	17.47 ± 0.55	2.41 ± 2.96	40.66 ± 1.96	8.35 ± 2.93
12	*Platycodon grandiflorus*	30.9 ± 3.54	14.93 ± 4.04	21.77	4.58 ± 1.50	0.79 ± 0.26	2.93 ± 2.93	1.52 ± 0.96
13	*Artemisia vulgaris*	181.9 ± 0.71	98.14 ± 1.52	5.32	24.91 ± 0.27	7.43 ± 1.44	79.68 ± 1	16.43 ± 3.91
14	*Ligustrum lucidum*	194.9 ±0.71	107.79 ± 0	12.02	29.1 ± 3.28	19 ± 0.78	108.42 ± 1.96	24.88 ± 0.98
15	*Scutellaria barbata*	188.4 ±1.41	93.5 ± 1.01	10.11	14.97 ± 2.73	9.26 ± 0.15	67.41 ± 3.91	20.62 ± 0.98

^1^DPPH free radical scavenging activity was measured in terms of equivalent of ascorbate (μM).

^2^Yeast oxidative stress was measured on the basis of survival of yeast cells (yeast growth) after treatment with H_2_O_2_.

^3^Total phenolics and flavonoid contents were expressed in gallic acid equivalent (GAE mg/g) and quercetin equivalent (QE mg/g) respectively.

^#^ Plants given in this table show significant correlation between their antioxidant activities and total phenolics / flavonoids contents. These plants are a subset of plants studied in this research (Table 1).

However, it should be noted that the antioxidant activities of the second group of plants did not show a good correlation between the antioxidant activity and their polyphenol content (Table 2). For example, *V. coloratum*, *H. diffusa*, *L. japonicus*, *A. paniculata* and *P. lactiflora* have displayed high antioxidant activity in both water and ethanol extracts, but contained low levels of phenolics and flavonoids. Similar findings were recently reported by the authors in a separate publication [6]. These observations demonstrate that, in addition to polyphenols, other constituents such as trace metals contribute to the antioxidant activities of medicinal plants. Indeed, a closer observation of the results for the second group of herbs (Tables 2 and 3) indicate that their antioxidant activities are due to the combination of polyphenols and trace metal contents. The results presented in Table 3, revealed that the second group of plants possess significant levels of trace metals. For instance, *V. coloratum*, has low levels of phenolics and flavonoids content but contains high levels of Zn, Mn and Mg (Table 3). This is in agreement with the literature that Zn and Mg play crucial role in antioxidant mechanisms [51-53]. Similarly, *H. diffusa*, *L. japonicus*, *A. paniculata* showed good antioxidant activity with high levels of Zn, Mg, Mn and Se. In many organisms, trace metals have been shown to act as co-factors of several antioxidant enzymes such as superoxide dismutase (SOD), peroxidases (POD), ascorbate peroxidase (APX) and other enzymes of ascorbate – glutathione pathway [54]. Therefore, the results obtained in this study strongly support trace metal involvement in antioxidant mechanisms. It is therefore hypothesized that the medicinal herbs display their antioxidant activities due to the combination of their total phenolics, flavonoids and the trace metal contents. Some of the plants investigated in this research possessed average levels of all these classes of antioxidants (phenolics, flavonoids and trace metals) and displayed significant antioxidant activities (Tables 2 and 3).

**Figure 2:**
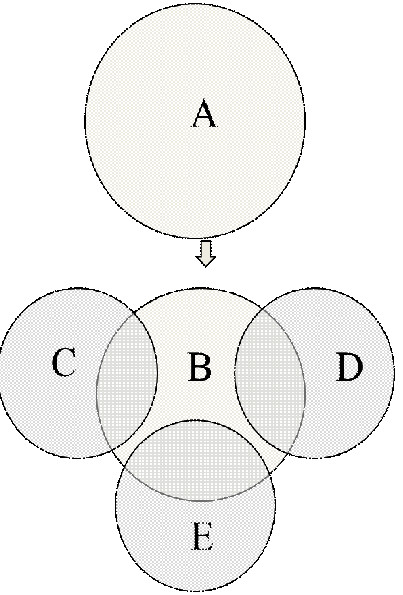
**A diagrammatic visualization scheme for the correlation of antioxidant activities and antioxidant content.** (**A**) Plants with high Antioxidant activity: 1, 2, 3, 4, 5, 6, 8, 10, 11, 13, 14, 15, 16, 18, 19, 20, 23, 26, 31, 33, 34, 36, 38, 39, 40, 41, 42, and 43; (**B**) Plants with high antioxidant activity and also contain significant antioxidant constituents: 1, 2, 3, 4, 5, 6, 8, 10, 13, 14, 15, 16, 18, 20, 23, 26, 31, 33, 34, 36, 38, 39, 40, 41, 42, and 43; (**C**) Plants with medium to high Phenolic content: 6, 13, 16, 20, 31, 33, 34, 41, 42, and 43; (**D**) Plants with medium to high Flavonoid content: 1, 4, 6, 8, 13, 16, 20, 29, 31, 33, 34, 36, 38, 41, 42, and 43; (**E**) Plants with medium to high Trace metal content: 2, 5, 10, 14, 15, 18, 23, 26, 27, 28, 31, 34, 37, 39, 40, 41, and 43. (Note: The names of the plants corresponding to the numbers in the legend are given in Table 1).

In order to obtain a comprehensive picture on the correlation of the observed activity and the content, all the active plants from Table 2 are considered and their activities are correlated in terms of the three active constituents (total phenolics, flavonoids and trace metals). A diagrammatic visualization scheme has been developed for this purpose which is presented in Figure 2. A brief description of this visualization scheme is given below.

All the plants with high DPPH scavenging activities (> 120 Ascorbate equivalent / g) have been included in the “Circle A” of Figure 2. However, the plants included in “Circle B” not only have high scavenging activity but also contain significant quantity of one or more antioxidant constituents (phenolics, flavonoids or trace metals). This automatically means that the plants in “Circle B” are a sub-set of plants in “Circle A”. Circles C, D and E represent the plants with medium to high quantities of phenolics, flavonoids and trace metals respectively (Tables 2 and 3). Any plant that exists in “Circle A” and also is present in one or more of the “Circles C, D or E” will be transferred into “Circle B”. Overlaps between antioxidant content circles (C, D, E) and antioxidant activity circle (B) represent the activities with respect to the corresponding constituents. As can be seen from Figure 2, the plants are active due to the presence of one or more out of the three active constituents. Some of the plants exhibit their activity due to the presence of all of the three antioxidant constituents (phenolics, flavonoids or trace metals). For instance, the plants *R. rubescens*, *S. officinalis, S. suberectus and U. rhyncophylla* have displayed their activities due to the presence of significant quantities of all the three antioxidant constituents (Figure 2). These findings further support the hypothesis that the medicinal herbs display their antioxidant activities due to the combination of their total phenolics, flavonoids and the trace metal contents. It should be noted here that only water extracts are considered in Figure 2.

It may be concluded from the above visualization scheme that the activities of 26 out of the 28 active plants could be explained in terms of their antioxidant content. Two of the plants, namely, *C. paniculatum* and *P. lactiflora* have displayed high antioxidant activities but did not contain significant quantities of any of the antioxidant constituents (Table 2 and Figure 2). One of the reasons for this non-correlation is likely to be due to the fact that some of the polyphenols may be extremely active owing to their structural characteristics even if they are present in smaller quantities [1]. Other reason includes the occurrence of antioxidant constituents (such as polysaccharides) that are not investigated in this study. Several studies demonstrated that botanical polysaccharides possess strong antioxidant activities [55-57].

Significant anti-inflammatory activities were observed for majority of the medicinal herbs studied here (Table 4). For instance, *S. officinalis*, *D. indica*, *P. suffuticosa*, *U. rhyncophylla* and *R. rubescens* have inhibited NO / TNF-α with low IC_50_ values and also contain high phenolics and flavonoids content. Bioactive molecules isolated in the literature from some of these plants showed significant anti-inflammatory properties [58,59]. For example, Sanguiin H-6 and H-11 isolated from *S. officinalis* has decreased the expression levels of iNOS [58]. Rhyncophylline and isorhyncophylline are isomeric alkaloids from *U. rhyncophylla* showed inhibition activity against the NO production and proinflammatory cytokines such as TNF-α and IL-1β production in LPS induced mouse N9 microglial cells [59].

## Conclusions

Forty-four selected medicinal plants have been investigated in this study for their antioxidant and anti-inflammatory activities. Amongst these plants, two distinct groups have been identified in terms of the correlation of antioxidant activities and their antioxidant contents. First group exhibited good relationship between total phenolics / flavonoids content and antioxidant activity, whilst in the second group such a relationship was poor. The observed biological activities of all the plants including those in the second group, could clearly be explained when trace metal content was considered together with polyphenols content. Amongst all the selected plants, *L. lucidum, P. suffuticosa, S. miltiorrhiza, S. officinalis, S. suberectus, T. farfara* and *U.rhyncophylla* showed significant antioxidant and anti-inflammatory activities with very low toxic effects. Consequently, the isolation of bioactive compounds from these target plants is underway in our laboratory.

## Competing interests

The authors declare that they have no competing interests.

## Authors' contributions

ASR, LZ, KS, MJU and MS have performed the experiments and analysis. ASR and LZ have contributed to the manuscript preparation. SRK and NR have designed the study, contributed to the analysis, critically evaluated the paper and provided the final manuscript. SJ helped with the preparation samples. PTS, BV, GM and JB have contributed to the manuscript preparation. All authors read and approved the final manuscript.

## Pre-publication history

The pre-publication history for this paper can be accessed here:

http://www.biomedcentral.com/1472-6882/12/173/prepub
